# Comparative analysis of the cardiomyocyte differentiation potential of induced pluripotent stem cells reprogrammed from human atrial or ventricular fibroblasts

**DOI:** 10.3389/fbioe.2023.1108340

**Published:** 2023-02-10

**Authors:** Lu Wang, Thanh Nguyen, Manuel Rosa-Garrido, Yang Zhou, David C. Cleveland, Jianyi Zhang

**Affiliations:** ^1^ Department of Biomedical Engineering, School of Medicine, School of Engineering, University of Alabama at Birmingham, Birmingham, AL, United States; ^2^ Department of Surgery, University of Alabama at Birmingham, Birmingham, AL, United States; ^3^ Children’s Hospital of Alabama, Birmingham, AL, United States; ^4^ Department of Medicine, Division of Cardiovascular Disease, School of Medicine, University of Alabama at Birmingham, Birmingham, AL, United States

**Keywords:** induced pluripotent stem cells, cardiac differentiation, action potential, field potential, calcium transient

## Abstract

**Background:** We had shown that cardiomyocytes (CMs) were more efficiently differentiated from human induced pluripotent stem cells (hiPSCs) when the hiPSCs were reprogrammed from cardiac fibroblasts rather than dermal fibroblasts or blood mononuclear cells. Here, we continued to investigate the relationship between somatic-cell lineage and hiPSC-CM production by comparing the yield and functional properties of CMs differentiated from iPSCs reprogrammed from human atrial or ventricular cardiac fibroblasts (^A^iPSC or ^V^iPSC, respectively).

**Methods:** Atrial and ventricular heart tissues were obtained from the same patient, reprogrammed into ^A^iPSCs or ^V^iPSCs, and then differentiated into CMs (^A^iPSC-CMs or ^V^iPSC-CMs, respectively) *via* established protocols.

**Results:** The time-course of expression for pluripotency genes (OCT4, NANOG, and SOX2), the early mesodermal marker Brachyury, the cardiac mesodermal markers MESP1 and Gata4, and the cardiovascular progenitor-cell transcription factor NKX2.5 were broadly similar in ^A^iPSC-CMs and ^V^iPSC-CMs during the differentiation protocol. Flow-cytometry analyses of cardiac troponin T expression also indicated that purity of the two differentiated hiPSC-CM populations (^A^iPSC-CMs: 88.23% ± 4.69%, ^V^iPSC-CMs: 90.25% ± 4.99%) was equivalent. While the field-potential durations were significantly longer in ^V^iPSC-CMs than in ^A^iPSC-CMs, measurements of action potential duration, beat period, spike amplitude, conduction velocity, and peak calcium-transient amplitude did not differ significantly between the two hiPSC-CM populations. Yet, our cardiac-origin iPSC-CM showed higher ADP and conduction velocity than previously reported iPSC-CM derived from non-cardiac tissues. Transcriptomic data comparing iPSC and iPSC-CMs showed similar gene expression profiles between ^A^iPSC-CMs and ^V^iPSC-CMs with significant differences when compared to iPSC-CM derived from other tissues. This analysis also pointed to several genes involved in electrophysiology processes responsible for the physiological differences observed between cardiac and non-cardiac-derived cardiomyocytes.

**Conclusion:** ^A^iPSC and ^V^iPSC were differentiated into CMs with equal efficiency. Detected differences in electrophysiological properties, calcium handling activity, and transcription profiles between cardiac and non-cardiac derived cardiomyocytes demonstrated that 1) tissue of origin matters to generate a better-featured iPSC-CMs, 2) the sublocation within the cardiac tissue has marginal effects on the differentiation process.

## Introduction

Despite the ongoing refinement of treatments for managing cardiovascular disease, long-term improvement is limited because adult mammals’ hearts cannot regenerate damaged myocardial tissue (Zhang et al., 2018). Whole-heart transplantation surgery remains the only proven option for treating patients who have entered the final stages of heart disease, but the availability of donated hearts is limited (Wang et al., 2021). Thus, researchers continue to develop strategies for repopulating the myocardial scar with exogenously administered cells; cardiomyocytes (CMs)—the fundamental contractile units of the heart—cannot be expanded in culture, so studies with human CMs generally cannot be conducted with cells obtained from primary sources.

The scarcity of CMs for therapeutic applications, as well as mechanistic studies and drug testing, was alleviated by the development of induced pluripotent stem cells (iPSCs), (Takahashi and Yamanaka, 2006), which are reprogrammed from cells of somatic tissues and, like embryonic stem cells (ESCs), can self-replicate indefinitely and be differentiated into cells of any lineage (Park et al., 2008a; Ebert et al., 2009; Soldner et al., 2009; Chen et al., 2018). iPSCs are typically generated *via* overexpression of four pluripotency factors (Oct3/4, Sox2, c-Myc, and klf4) (Takahashi and Yamanaka, 2006) and can be reprogrammed from a wide variety of cell types (Aasen et al., 2008; Aoi et al., 2008; Hanna et al., 2008; Kim et al., 2008; Stadtfeld et al., 2008; Utikal et al., 2009; Polo et al., 2010; Sanchez-Freire et al., 2014). Reprogrammed cells retain some of the epigenetic characteristics associated with their somatic-cell lineage, and this “epigenetic memory” appears to influence both the yield and functional properties of iPSC-derived cells (Kim et al., 2011; Rizzi et al., 2012; Meraviglia et al., 2016). We had shown that CMs were more efficiently differentiated from human iPSCs (hiPSCs) when the hiPSCs were reprogrammed from cardiac fibroblasts (^hCF^iPSCs) rather than dermal fibroblasts (^hDF^iPSCs) or umbilical cord blood mononuclear cells (^hUCB^iPSCs). ^hCF^iPSCs -derived CMs also have a more cardiac-like Ca^2+^ handling profile, (Zhang et al., 2015), suggesting that hiPSCs may be more suitable for generating hiPSC-CMs if they are reprogrammed from cardiac, rather than non-cardiac lineage cells. The experiments described in this report continue to interrogate the relationship between the somatic-cell lineage of hiPSCs and hiPSC-CM production by comparing the yield and functional properties of CMs differentiated from hiPSCs reprogrammed from atrial cardiac fibroblasts or ventricular cardiac fibroblasts (^A^iPSC-CM or ^V^iPSC-CM, respectively).

## Materials and methods

All protocols in this study were approved by the Institutional Review Board (IRB) for Human Use at the University of Alabama, Birmingham.

### Isolation and characterization of cardiac fibroblasts

Cardiac ventricular and atrial tissue specimens were obtained with informed consent from a 15-day-old patient with d-TGA (dextro-Transposition of the Great Arteries) severe pulmonary outflow obstruction who underwent open chest surgery; then, cardiac fibroblasts were isolated as described previously (Park et al., 2008b). Briefly, tissue specimens were cut into small pieces, placed in a 6-well plate containing hFib media (DMEM containing 10% FBS, 2 mM L-GIn, 50 U ml^-1^ penicillin and 50 mg mL^-1^ streptomycin), and covered with a cover slip. The culture medium was changed every three days for 2 weeks, and fibroblast outgrowth was observed after 10 days of culture. Fibroblast identification was confirmed *via* immunofluorescence staining for vimentin and TE-7 expression (Gao et al., 2018).

### Immunofluorescence staining

Immunofluorescence staining was performed as previously described (Wang and Zhang, 2022). Briefly, cells were fixed in 4% paraformaldehyde (PFA) for 15 min at room temperature, permeabilized with 90% acetone for 3 min, and then blocked with 10% donkey serum for 20 min. The fixed cells were incubated with primary antibodies (Supplementary Table S1) overnight at 4°C and with corresponding fluorescently conjugated secondary antibodies at room temperature for 2 h; then, the cells were mounted with mounting medium containing 4,6-diamidino-2-phenyl- indole (DAPI) (Vector Laboratories; H-1200) and imaged with a confocal microscope.

### iPSC reprogramming

iPSC reprogramming was performed with a CytoTune™-iPS 2.0 Sendai Reprogramming Kit as directed by the manufacturer’s instructions. Briefly, cells (passage 2) were transduced with CytoTune™-iPS 2.0 Sendai reprogramming vectors and then transferred to feeder cells in fibroblast medium (DMEM supplemented with 10% FBS, 1% MEM Non-Essential Amino Acids Solution, and 0.1% 2-mercaptoethanol). Putative hiPSC colonies were identified *via* Tra1-60 live staining, transferred into Matrigel-coated wells containing mTeSR medium, and expanded without feeder cells.

### iPSC characterization

OCT4, Nanog, SSEA4, and SOX2 expression in reprogrammed iPSCs was evaluated *via* immunofluorescence staining. The absence of residual Sendai virus was confirmed after 10 passages by qRT-PCR with a primer for SeV amplification as previously described; (Grossmann et al., 2021); RNA from passage 0 iPSCs and from somatic fibroblasts served as the positive and negative controls, respectively. The absence of *mycoplasma* was confirmed at passage 12 with a LookOut *Mycoplasma* PCR Detection Kit (Sigma-Aldrich—Merck) as directed by the manufacturer’s instructions. The PCR products were loaded on a 1% agarose gel containing SYBR Safe DNA Gel Stain (Invitrogen, S33102) and visualized with a ChemiDoc MP Imaging System (Bio-Rad).

### Teratoma formation

The teratoma formation assay was performed as described previously (Nelakanti et al., 2015). Briefly, ∼1 × 10^6^ iPSCs were collected with Accutase, suspended in 50 μL Matrigel, and slowly injected into the gastrocnemius muscle of NOD/SCID Gamma mice. Teratomas were monitored and surgically removed 6–8 weeks after injection, fixed with 4% formaldehyde, and embedded in paraffin. The preserved samples were sectioned and stained with hematoxylin and eosin at the Pathology Core Research Laboratory in the Department of Pathology, University of Alabama, Birmingham.

### Karyotype analysis

Karyotype analysis was conducted in the Cytogenetics Lab at the WiCell Research Institute (Madison, WI). Metaphases were analyzed for each sample by using a brightfield microscope after G-banding. Chromosome identification and karyotype descriptions were made according to the International System for Human Cytogenetic Nomenclature (McGowan-Jordan, 2016). Short tandem repeat (STR) analysis was performed in the Histocompatibility/Molecular Diagnostics laboratory at the University of Wisconsin Hospital and Clinics (Madison, WI).

### Cardiomyocyte differentiation from ^A^iPSC and ^V^iPSC

^A^iPSC and ^V^iPSC were differentiated into cardiomyocytes (CMs) as previously described (Lian et al., 2013). Briefly, ^A^iPSC and ^V^iPSC were maintained with mTeSR™ Plus (Stem Cell Technologies) cell media in GelTrex-coated (Thermo Fisher Scientific) plate until meeting 80% confluency. Cardiomyocyte differentiation was induced by culturing iPSCs with CHIR99021 for 24 h in RPMI 1640 medium and B27 without insulin (B27-) media. Cells were then recovered for 48 h in RPMI 1640 medium and B27- media, and cultured with IWR-1 for 48 h in RPMI 1640 medium and B27- media. Beating cardiomyocytes typically appeared nine days after differentiation initiation.

### Quantitative real-time polymerase chain reaction (qRT-PCR)

Total RNA was extracted using RNeasy mini kits (Qiagen, United States) as directed by the manufacturer’s instructions and quantified *via* Nanodrop. cDNA was synthesized with SuperScriptTM II Reverse Transcriptase (Thermo Scientific, United States), and qRT-PCR was performed on a QuantStudio three real-time PCR system (Eppendorf, United States) with appropriate primers (Supplementary Table S2) and the Power Up SYBR Green PCR Mix (Thermo Fisher Scientific). Measurements were determined *via* the 2^−ΔΔCT^ method and normalized to the abundance of glyceraldehyde phosphate dehydrogenase (GAPDH) RNA.

### Flow cytometry

Purity of differentiated hiPSC-CMs was determined *via* flow cytometry analysis as previously described (Gao et al., 2020). Briefly, iPSC-CMs were trypsinized into single cells, fixed with fixation and permeabilization solution (51-2090KZ) for 30 min at 4°C, and blocked in Human BD Fc Block (564219) at room temperature for 10 min. Cells were then incubated with primary antibodies and isotype control antibodies at room temperature for 40 min, incubated with corresponding fluorescently conjugated secondary antibodies for 30 min, and resuspended in wash buffer (554723). Flow cytometry was performed with an LSR Fortessa instrument (BD Biosciences, United States).

### Multi-electrode array (MEA) analysis

hiPSC-CM action and field potentials were assessed with a MaestroEdge multi-electrode array system as previously described (Wickramasinghe et al., 2022). Briefly, cells were plated on 24-well CytoView MRA plates (Axion BioSystems) coated with GelTrex at a density of 4 × 10^4^ cells per well. One week later, the plates were equilibrated for 10 min, data were recorded using the Axion Integrated Studio (AxIS) software and finally analyzed using the Cardiac Analysis Tool (Axion Biosystems, Atlanta, GA, United States).

### Ca^2+^ transient analysis

Ca^2+^ transients were measured as previously described (Gao et al., 2020). Briefly, hiPSC-CMs were plated as individual cells on cover glasses (25 × 25 mm) coated with Geltrex and incubated with Fura-2 AM (0.5 μM, Invitrogen, United States) for 10 min in Tyrode’s solution. Cells were then stimulated at 1 Hz and 2 Hz, and the ratio of fluorescence emitted at 340 and 380 nm was determined with a Ca^2+^ recording system and analyzed using IonWizard (IonOptix, United States) software.

### Generating the bulk-RNA sequencing data

^A^iPSC, ^V^iPSC, ^A^iPSC-CM, and ^V^iPSC-CM RNA sequencing was performed by Novogene—Advancing Genomics using Illumina NovaSeq 6,000 platforms. RNA quality control was achieved by removing reads containing adapters, having more than 10% of undetermined bases, and low-quality reads. Less than 2% of these reads were removed in all samples. Reads were mapped to human GRCh38 reference genome (Nurk et al., 2022) using STAR pipeline (Dobin et al., 2013); the unique mapping rate was above 94% in all samples (Supplementary Table S3). Then, for each gene, the count of transcripts for each sample was recorded.

### Collecting iPSC-CM bulk-RNA sequencing reported in the literature

The phrase “hiPSC derived cardiomyocyte” was used to search for iPSC-CM sequencing data in Gene Expression Omnibus database (https://www.ncbi.nlm.nih.gov/geo/); this query yields 69 data sources. Publications associated with these data sources were manually read to select data sets that 1) had the same cardiomyocyte differentiation (from iPSC) protocol as ours, without further chemical or genetic modification treatment; 2) iPSC-CMs were harvested 30 days or later after differentiation; 3) reported iPSC-CM electrophysiology; 4) have the iPSC-CM bulk-RNA sequencing data generated by Illumina platforms; and 5) the transcript raw count format (required for DeSeq2 gene expression normalization) was available. Two datasets (Zhao et al., 2017; Garay et al., 2022) were selected to be analyzed with ^A^iPSC, ^V^iPSC, ^A^iPSC-CM, and ^V^iPSC-CM RNA sequencing data, which were summarized in Supplementary Table S4. Dataset (Garay et al., 2022) contains iPSC-CM derived from skin tissue after 90 days of differentiation from skin (^S−D90^iPSC-CM) and kidney tissue (^K−D90^iPSC-CM). Dataset (Zhao et al., 2017) contains iPSC derived from the dermal fibroblast (skin) tissue (^S^iPSC) and iPSC-CM after 30 days of differentiation (^S−D30^iPSC-CM).

#### Bulk-RNA gene expression analysis

The raw transcript counts (35,794 genes) in each sample (24 samples) were normalized by Deseq2 (Love et al., 2014). After normalization, the sample pairwise similarities were calculated using all samples’ expression data; then, the samples were clustered by applying hierarchical clustering (clustergram) (Frank, 2016; clustergram, 2022) on these similarities. Also, the normalized expression was embedded and visualized by Uniform Manifold Approximation (UMAP) toolkit (Leland McInnes and James, 2018; Uniform, 2021).

The expression fold-change was calculated for each gene to select differentially expressed genes (DEG) in two-group comparison. Due to low sample size (n = 3), the non-parametric Wilcoxon Ranksum test was implemented and applied as in (Bian et al., 2021) for statistical analysis. Genes with fold-change magnitude of two and above (>2 or < 0.5), average per-sample expression of 100 and above, and *p*-value < 0.05 were selected as DEGs.

#### Gene ontology analysis

The list of DEG, computed in bulk-RNA data analysis, was input into The Database for Annotation, Visualization and Integrated Discovery (DAVID) (Huang et al., 2009). DAVID resulted in a list of gene ontologies and signaling pathways enriched by the input DEG. Only, gene ontologies and signaling pathways with a False Discovery Rate of 0.05 and below were retained for statistical significance. The enrichment score was computed as the log-base-10 of the enrichment *p*-value, which was resulted from DAVID.

#### Statistical analysis

Data were presented as mean ± SEM. Significance was determined *via* the Student’s t-test for comparisons between two groups and *via* one-way analysis of variance for comparisons among three or more groups. A *p* value of <0.05 was considered statistically significant.

For RNA sequencing data, due to the small sample size, non-parametric Wilcoxon-Ranksum test was applied for comparison between two groups, and non-parametric Kruskal–Wallis test was used for comparison among three or more groups. When a large number of genes were tested in the Deseq2 protocol, the Benjamini and Hochberg method was applied to adjust the *p*-values (false-positive correction). A *p*-value of < 0.05 was considered statistically significant.

## Results

### Characterization of iPSCs reprogrammed from human atrial and ventricular fibroblasts

**FIGURE 1:**
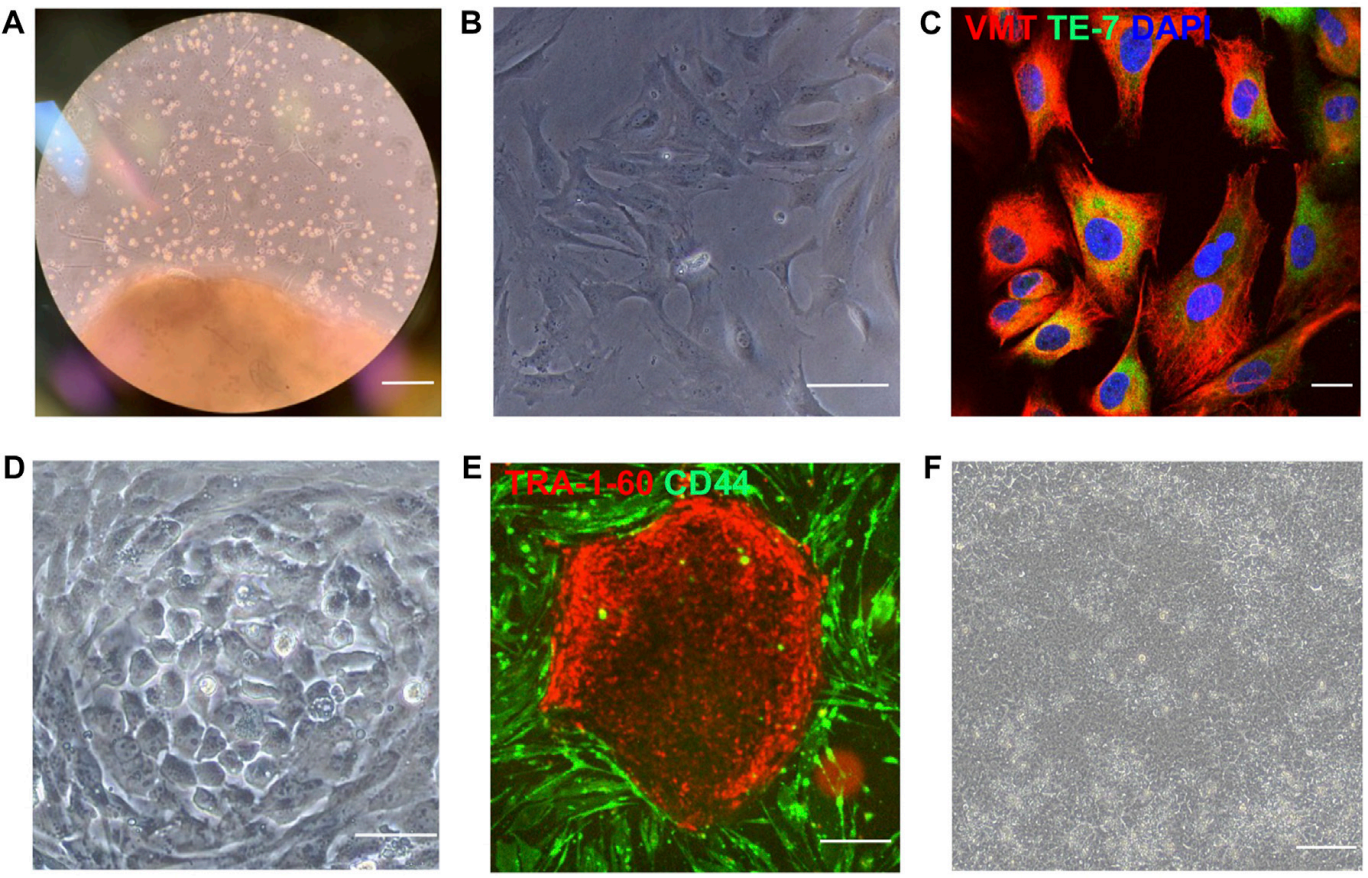
iPSCs were reprogrammed from human atrial and ventricular fibroblasts. **(A)** Cardiac tissue was obtained from the atrium and ventricle of a male newborn infant patient who underwent open-chest surgery for d-TGA and cultured to induce fibroblast outgrowth. **(B)** Isolated fibroblasts (bar = 100 μm) were **(C)** evaluated for expression of the fibroblast-specific markers vimentin (VMT) and TE-7 *via* immunofluorescence staining (bar = 20 μm) and then reprogrammed **(D)** into ^A^iPSC and ^V^iPSC *via* transfection with Sendai virus coding for OCT4, SOX2, KLF4, and C-MYC (bar = 100 μm). **(E)** Three weeks after transduction, putative hiPSCs were identified *via* live immunofluorescent staining for CD44 and the pluripotency marker Tra1-60 (bar = 200 μm) and **(F)** imaged for morphological assessments (bar = 100 μm). Representative images of cultured ventricular tissue (Panel A), ventricular fibroblasts **(B–C)**, and ^V^iPSC **(D–F)** are displayed.

**FIGURE 2:**
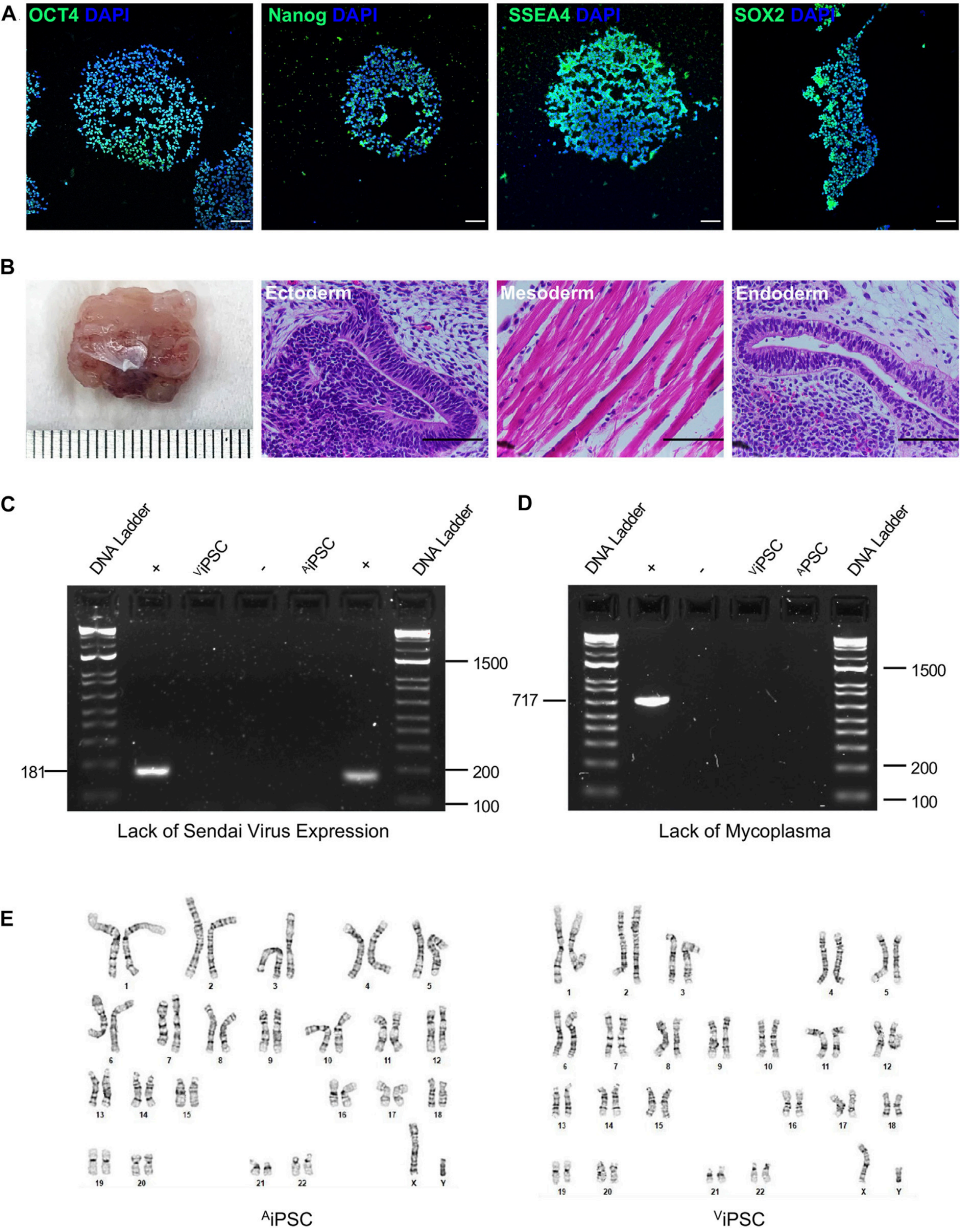
^A^iPSC and ^V^iPSC were pluripotent with normal karyotypes. **(A)** Expression of the pluripotency markers OCT4, Nanog, SSEA4, and Sox2 was evaluated *via* immunofluorescence staining in ^A^iPSC and ^V^iPSC. Nuclei were counterstained with DAPI (representative images of ^V^iPSC; bar = 100 μm) **(B)** ^A^iPSC and ^V^iPSC were subcutaneously transplanted into immunodeficient mice and grew to form teratomas over the ensuing 8 weeks; then, the teratoma was excised, sectioned, stained with hematoxylin and eosin, and examined for the presence of all three developmental germ layers: ectoderm (i.e., neural epithelium), mesoderm (i.e., striated muscle), and endoderm (i.e., gut-like epithelium) (representative images for ^V^iPSC teratomas; bar = 100 μm). **(C)** Loss of the Sendai virus vector was confirmed by electrophoresis of RT-PCR products at passage 10. **(D)** The absence of *mycoplasma* contamination was confirmed at passage 12 by electrophoresis of RT-PCR products from the culture supernatant. **(E)** Karyotype analyses were performed to confirm correct chromosomal number and structure of the selected cells.

Both atrial and ventricular heart tissues were obtained from the same male newborn infant patient during open-chest surgery for d-TGA and cut into small pieces to induce fibroblast outgrowth (Figure 1A); then, atrial and ventricular fibroblasts were collected, expanded for two weeks (Figure 1B), and stained for the expression of vimentin and TE-7 to confirm fibroblast identity (Figure 1C). Fibroblasts were reprogrammed into ^A^iPSC and ^V^iPSC *via* transfection with non-integrating Sendai virus vectors coding for expression of the human variants of Oct3/4, Sox2, Klf4, and c-Myc. Two weeks later, colonies with an ESC-like morphology (Figure 1D) expressing the pluripotent/stem-cell marker Tra1-60 (Figure 1E) were mechanically isolated and expanded. The morphologies of the ^A^iPSC and ^V^iPSC lineages were indistinguishable (Figure 1F). Both ^A^iPSC and ^V^iPSC expressed the pluripotency genes OCT4, Nanog, SSEA4, and SOX2 (Figure 2A) and generated cells from all three germ layers when evaluated *via* teratoma formation assay (Figure 2B). Similar results for ^A^iPSC see Supplementary Figure S1. Sendai virus (Figure 2C) and *mycoplasma* (Figure 2D) were undetectable after 10 and 12 passages, respectively, and the cells’ karyotypes were normal (Figure 2E). (Grossmann et al., 2021)

### CM differentiation potential of ^A^iPSC and ^V^iPSC

**FIGURE 3:**
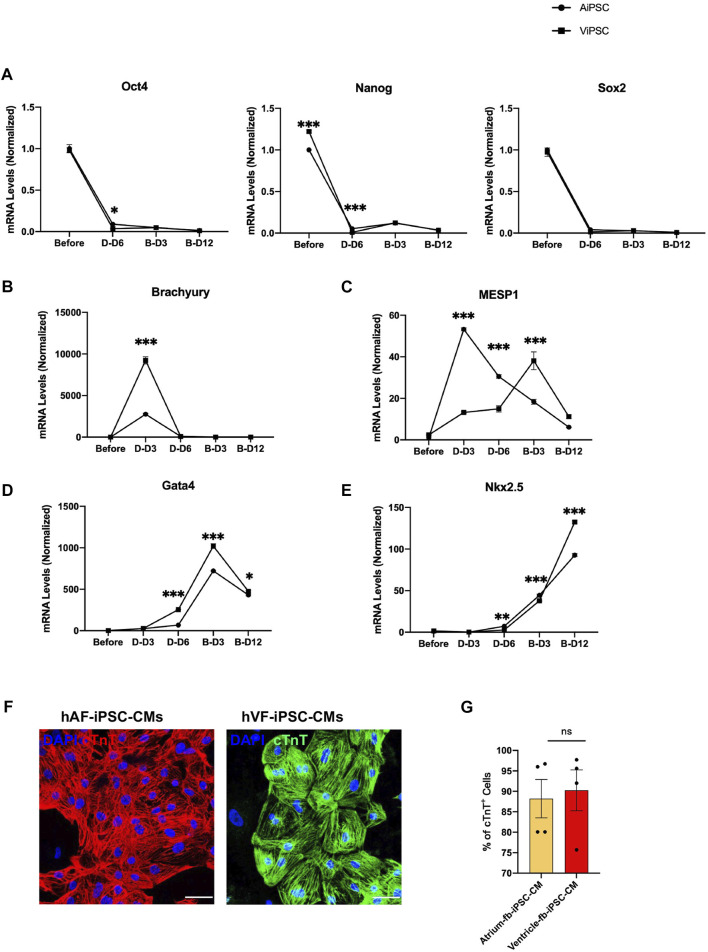
Differentiation of ^A^iPSC and ^V^iPSC into CMs was equally efficient. ^A^iPSC and ^V^iPSC were differentiated into ^A^iPSC-CM and ^V^iPSC-CM *via* established protocols. The abundance of mRNA for **(A)** the pluripotency genes Oct4, Nanog, and Sox2; **(B)** the early mesoderm marker Brachyury, **(C)** the early cardiac-mesoderm marker Mesp1; and the cardiac-cell markers **(D)** Gata4 and **(E)** Nkx2.5 was evaluated before differentiation, 3 days (D-D3) and 6 days (D-D6) after differentiation was initiated, and 3 days (B-D3) and 12 days (B-D12) after beating was observed. Measurements were performed *via* qRT-PCR and normalized to the abundance of glyceraldehyde phosphate dehydrogenase mRNA **(F)** ^A^iPSC-CM and ^V^iPSC-CM were immunofluorescently stained for the expression of cardiac troponin T (cTnT); bar = 50 μm. **(G)** The purity of the ^A^iPSC-CM and ^V^iPSC-CM was quantified *via* flow cytometry analyses of cTnT-expressing cells on B-Day 12. *p < 0.05, **p < 0.01, ***p < 0.001.

mRNA assessments of the expression of pluripotency genes (OCT4, NANOG, and SOX2; Figure 3A), the early mesodermal marker Brachyury (Figure 3B), the cardiac mesodermal markers MESP1 (Figure 3C) and Gata4 (Figure 3D), and the cardiovascular progenitor-cell transcription factor NKX2.5 (Figure 3E) were conducted before the ^A^iPSC and ^V^iPSC were differentiated into hiPSC-CMs (Day 0), three and six days after the differentiation protocol (Lian et al., 2012) was initiated (D-Day 3 and D-Day 6), and 3 and 12 days after the cells began beating (B-Day 3 and B-Day 12). Measurements in both hiPSC lineages indicated that pluripotency gene expression declined from Day 0 to D-Day 6. At the same time, the abundance of Brachyury and Gata4 mRNA peaked on D-Day 3 and B-Day 3, respectively, and NKX2.5 expression progressively increased from D-Day 6 through B-Day 12. However, whereas measures of MESP1 abundance gradually increased through B-Day 3 in ^V^iPSC, MESP1 expression in ^A^iPSC peaked, and was significantly greater than in ^V^iPSC, on D-Day 3. Nevertheless, when the expression of cardiac troponin T (cTnT) (Figure 3F) was evaluated *via* immunofluorescence, the proportions of positively stained ^A^iPSC-CM and ^V^iPSC-CM populations were similar (Figure 3G) on B-Day 12, indicating that the differentiation protocol was equally efficient for both hiPSC lines.

### Functional properties of ^A^iPSC-CM and ^V^iPSC-CM

**FIGURE 4:**
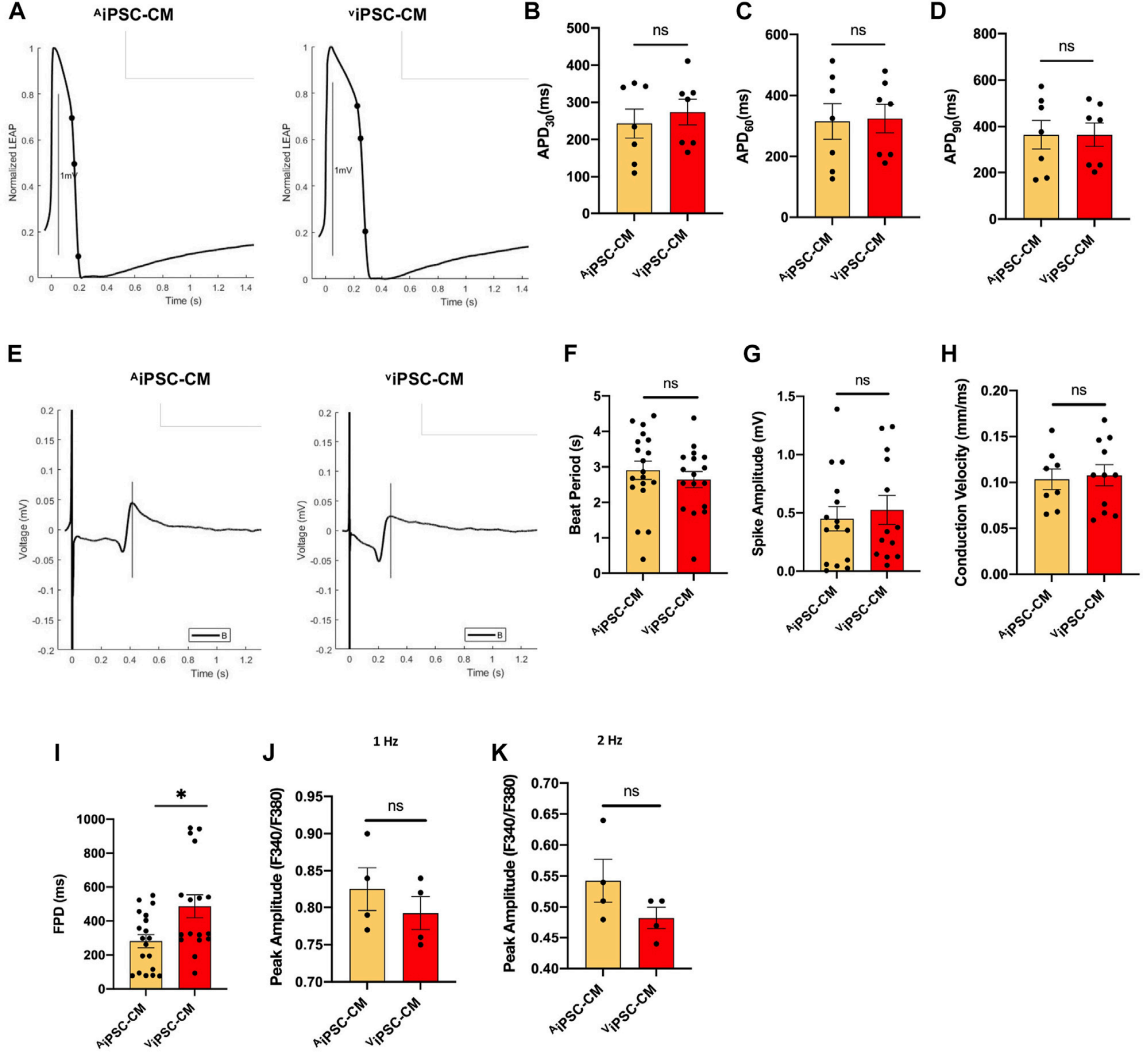
Electrophysiological properties of ^A^iPSC-CM and ^V^iPSC-CM were similar. **(A)** Representative action-potential and **(E)** field-potential recording are displayed for ^A^iPSC-CM and ^V^iPSC-CM. **(B–D)** Action potential durations to 30% (APD_30_), 60% (APD_60_), and 90% (APD_90_) recovery were calculated for ^A^iPSC-CM and ^V^iPSC-CM. **(F–I)** Field potential recordings were used to calculate **(F)** beat period, **(G)** spike amplitude, **(H)** conduction velocity, and **(I)** field-potential duration (FPD) for ^A^iPSC-CM and ^V^iPSC-CM. **(J,K)** Peak Ca^2+^ transient amplitudes were calculated from Fura-2 AM dye fluorescence recordings in ^A^iPSC-CM and ^V^iPSC-CM while the cells were paced at 1 Hz and 2 Hz *p < 0.05.

Electrophysiological properties of ^A^iPSC-CM and ^V^iPSC-CM were characterized *via* action potential (Figure 4A) and field potential (Figure 4E) recordings acquired 30 days after the differentiation protocol was initiated. Action potential durations to 30% (APD_30_, Figure 4B), 60% (APD_60_, Figure 4C), and 90% (APD_90_, Figure 4D) recovery, as well as measurements of beat period (Figure 4F), spike amplitude (Figure 4G), and conduction velocity (Figure 4H) were nearly identical in ^A^iPSC-CM and ^V^iPSC-CM. Field-potential durations (Figure 4I) were detected to be significantly longer in ^V^iPSC-CM. Calcium transients tended to have higher peak amplitudes in ^A^iPSC-CM than in ^V^iPSC-CM when the cells were paced at 1 or 2 Hz (Figures 4J,K), but the differences between groups did not reach statistical significance.

On the other hand, Table 1 shows that our ^A^iPSC-CM and ^V^iPSC-CM APD_90_ (measured at 1 Hz) were within the range of the iPSC-CM derived from the reported skin, kidney, and peripheral blood tissues. Meanwhile, ^A^iPSC-CM/^V^iPSC-CM had significantly higher conduction velocity (Table 2) than iPSC-CM derived from other tissues.

**TABLE 1:** Comparison of ADP_90_ (in ms) between ^A^iPSC-CM/^V^iPSC-CM and other reported iPSC-CM derived from skin, kidney, and peripheral blood tissues. The numbers were written in mean (standard deviation) format.

Cell line	Tissue origin	ADP_90_, in ms
^A^iPSC-CM	Cardiac	390 (200)
^V^iPSC-CM	Cardiac	390 (190)
Lan et al. (2020)	Skin	200 (20) (*)
Poulin et al. (2021)	Skin	400 (50)
Guo et al. (2019)	Skin	378.9 (17.2)
Garay et al. (2022) (^S−D90^iPSC-CM and ^K−D90^iPSC-CM)	Skin (dermal fibroblast)/Kidney (HEK293 cell)	176 (7)
Lam et al. (2020)	Peripheral blood	290 (40) (**)
Wang et al. (2022)	Peripheral blood	400 (50) (***)

(*): The precise numbers in Lan et al. (2020) are not reported. The table numbers were estimated from Lan et al. (2020) Figure 1.

(**): The precise numbers in Lam et al. (2020) are not reported. The table numbers were estimated from Lam et al. (2020) Figure 6.

(***): The precise numbers in Wang et al. (2022) are not reported. The table numbers were estimated from Wang et al. (2022) Figure 4.

**TABLE 2:** Comparison of conduction velocity (in cm/s) between ^A^iPSC-CM/^V^iPSC-CM and other reported iPSC-CM derived from skin, kidney, and peripheral blood tissues. The numbers were written in mean (standard deviation) format.

Cell line	Tissue origin	Conduction velocity
^A^iPSC-CM	Cardiac	10.3 (0.5)
^V^iPSC-CM	Cardiac	10.7 (0.5)
Poulin et al. (2021)	Skin	9.1 (0.7)
Zhao et al. (2017) (^S−D30^iPSC-CM)	Skin	7.9 (0.2) (*)
Garay et al. (2022) (^S−D90^iPSC-CM and ^K−D90^iPSC-CM)	Skin (dermal fibroblast)/Kidney (HEK293 cell)	4.5 (4)
Lam et al. (2020)	Peripheral blood	6 (2) (**)
Riedel et al. (2014)	Peripheral blood	4.8 (0.6) (***)
Ong et al. (2017)	Peripheral blood	4.3 (0.2) (****)

(*): The precise numbers in Zhao et al. (2017) are not reported. The table numbers were estimated from Zhao et al. (2017) Supplementary Figure S2.

(**): The precise numbers in Lam et al. (2020) are not reported. The table numbers were estimated from Lam et al. (2020) Figure 6.

(***): The precise numbers in Riedel et al. (2014) are not reported. The table numbers were estimated from Riedel et al. (2014) Figure 5.

(****): The precise numbers in Ong et al. (2017) are not reported. The table numbers were estimated from Ong et al. (2017) Figure 5.

### Transcriptional heterogeneity among iPSC and iPSC-CM samples

**FIGURE 5:**
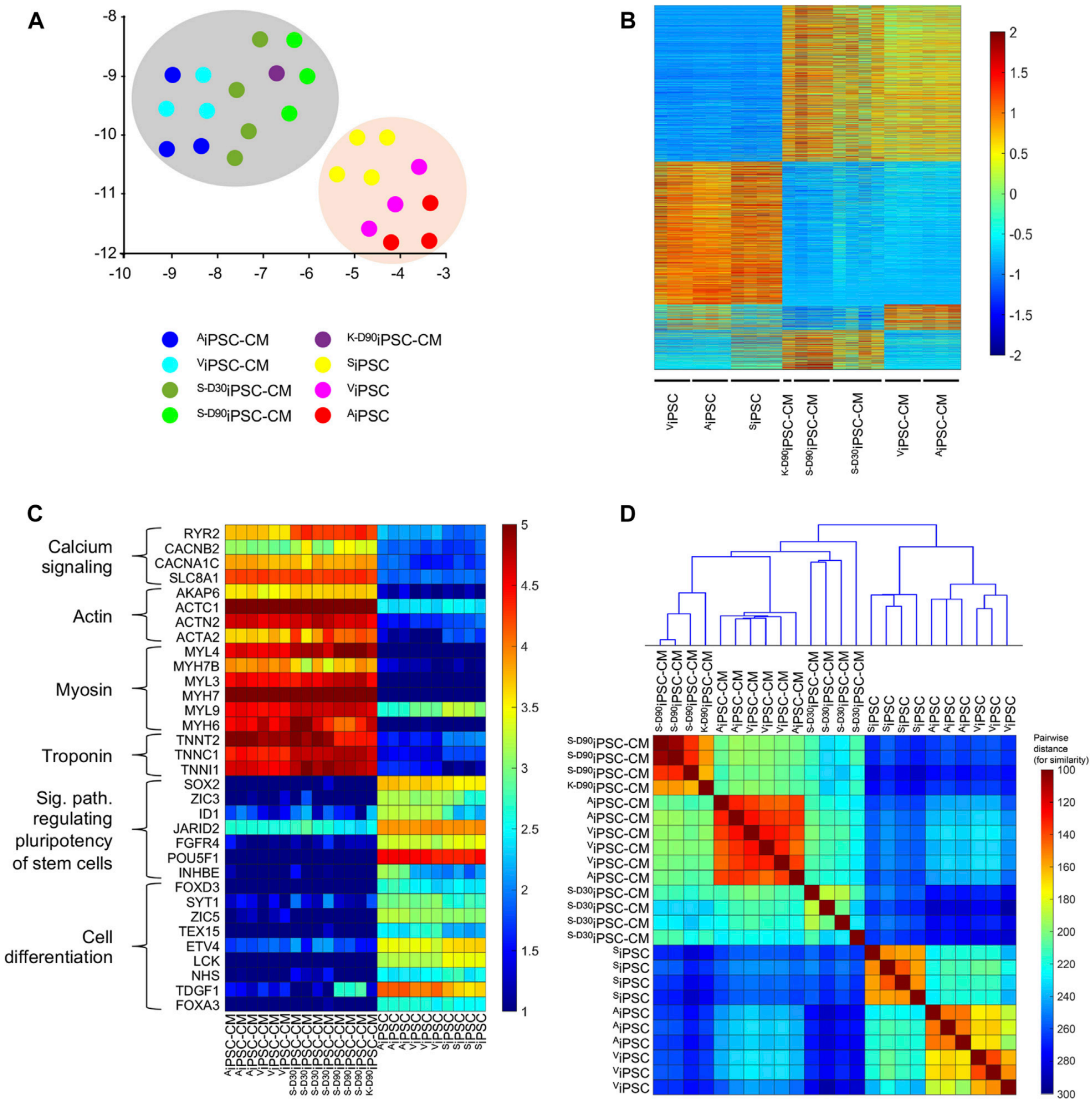
Transcriptional analysis of iPSC and iPSC-CM cell lines. **(A)** UMAP plot, where the entire gene expression (35,794 genes) was embedded and reduced to two dimensions. Note the difference in the transcriptional profile between iPSC-CM (grey shade) and iPSC (orange shade). **(B)** Heatmap of differentially expressed genes (*p*-value <0.05) when comparing iPSC to iPSC-CM and among iPSC-CM from different origins. While a high number of differentially expressed genes were detected when comparing iPSC and iPSC-CM samples (4,970), a mild number was observed between ^A^iPSC-CM/^V^iPSC-CM and other iPSC-CM samples and almost non-significative differences were identified after comparing ^A^iPSC-CM and ^V^iPSC-CM (124). The colors represent the z-score normalization (gene expression magnitude is ignored). Red color denotes upregulation of transcripts, while blue denotes downregulation of mRNA levels. **(C)** Heatmap of selected gene to show the efficiency of the iPSC-CM differentiation process. Calcium signaling, Actin, Myosin, Troponin, signaling pathway regulating stem cell pluripotency, and cell differentiation gene expression markers are differentially expressed among the iPSC-CM and iPSC cell lines. The colors represent the normalized expression *via* Deseq2 pathway to emphasize the expression magnitude of these genes. **(D)** Clustergram plot including a heatmap of pairwise Euclidian distance among the 24 studied samples and the cluster hierarchy among these. Note the small distance (high similarity) between ^A^iPSC-CMs and ^V^iPSC-CMs compared to the rest of the iPSC-CMs and iPSC.

RNA-seq experiments were performed to compare the transcriptional profiles of iPSC and derived cardiomyocytes from this study (^A^iPSC-CM, ^V^iPSC-CM) and two works where iPSC were generated and differentiated to cardiomyocytes using skin (^SD30^iPSC-CM, ^S−D90^iPSC-CM) and kidney fibroblast (^K−D90^iPSC-CM) (Zhao et al., 2017; Garay et al., 2022). Uniform Manifold Approximation and Projection (UMAP) analysis of the data showed clear separation of cardiomyocytes and their original induced pluripotent stem cells, suggesting that the differentiation process was successful in all cases (Figure 5A). Transcriptional analysis comparing derived cardiomyocytes and iPSC identified 4,970 differentiated expressed genes between both groups (Figure 5B). When focused on this difference, a significant upregulation of genes belonging to the calcium signaling, Actin, Myosin, and Troponin in cardiomyocytes was detected in iPSC-CM. iPSC samples also showed upregulation of genes related to signaling pathways regulating stem cell pluripotency and cell differentiation (Figure 5C). Comparison between ^A^iPSC-CM/^V^iPSC-CM (derived from cardiac tissue), ^S−D30^iPSC-CM/^S−D90^iPSC-CM (derived from skin tissue) and ^K−D90^iPSC-CM (derived from kidney) revealed minor transcriptional differences between cardiomyocytes derived from heart tissue (124 genes differentially expressed) and moderated change when compared to those differentiated from skin or kidney (1,081 differentially expressed genes) (Figure 5B). A clustergram to study the pairwise similarity among the iPSC and iPSC-CM samples demonstrated that while the detected transcriptional changes are mild, these are enough to establish significant differences between heart and non-heart tissue differentiated cardiomyocytes. iPSC and derived cardiomyocytes form two very different clusters. When focusing only on derived cardiomyocytes, clear subclusters can be observed separating cardiac-tissue-derived cardiomyocytes (^A^iPSC-CM and ^V^iPSC-CM) from ^S−D30^iPSC-CM, ^S−D90^iPSC-CM and ^K−D90^iPSC-CM (Figure 5D). Gene ontology analysis of the 1081 DE genes (fold change >2 see method section) identified 44 terms, some of them related to heart function (Supplementary Table S5). Further analysis identified 12 genes (AKAP9, BIN1, CACNA1G, CACNB2, CAV1, DSP, GJA5, IRX3, NKX2-5, RANGRF, RYR2 and ISL1) involved in the electrical conduction system of the heart (Zhou et al., 2011) (Supplementary Figure S3), suggesting that the differential expression of these genes play a key role in explaining the distinct electrophysiological properties detected between cardiac and non-cardiac differentiated cardiomyocytes (Tables 1,2). Further analysis between ^A^iPSC-CM and ^V^iPSC-CM samples revealed no significant difference in gene expression for atrial (HEY2 and MYL2), ventricular (MYL7 and NPPA), and pacemaker (HCN4, TBX3, and GJC1) cardiomyocyte markers (Supplementary Figure S4).

## Discussion

We have shown that when hiPSCs were reprogrammed from cardiac fibroblasts (^hCF^iPSCs), dermal fibroblasts (^hDF^iPSCs), or umbilical cord blood mononuclear cells (^hUCB^iPSCs) and then differentiated into sheets of CMs, spontaneous beating was observed in ≥ 90% of all ^hCF^iPSC-CM batches tested, but fell to as low as 20%-30% of ^hDF^iPSCs-CM and ^hUCB^iPSCs-CMs (Zhang et al., 2015). Notably, the fibroblasts used for ^hCF^iPSCs generation were obtained from the atrium, and more than 200 genes are differentially expressed between atrial and ventricular fibroblasts (Burstein et al., 2008). Thus, we investigated whether the transcriptional differences between atrial and ventricular fibroblasts may be accompanied by variations in the differentiation potential of hiPSCs reprogrammed from the two fibroblast subtypes. The results from our studies indicate that ^A^iPSC-CM and ^V^iPSC-CM can be differentiated into CMs with equal efficiency. Although field potential durations were significantly longer for ^V^iPSC-CM than ^A^iPSC-CM, measurements of action-potential duration, spike amplitude, conduction velocity, and calcium handling were essentially indistinguishable. Thus, the CM differentiation potential of ^A^iPSC and ^V^iPSC appears not significantly different.

In this work, our generated iPSC-CM derived from cardiac fibroblast showed higher conduction velocity than previously reported iPSC-CM derived from the skin and peripheral blood tissues**.** However, comparing iPSCs derived from ventricular fibroblasts and atrial fibroblasts from the same donor showed no significant electrophysiological difference between iPSCs derived from ventricular fibroblasts and atrial fibroblasts, except in field potential duration. Also, we evaluated the gene expression of representative markers during cardiomyocyte differentiation and measured the purity of cardiomyocytes; the results demonstrated no significant difference.

This report examined gene expression profile differences among the iPSC-CM cell lines. Overall, the difference between ^A^iPSC-CM and ^V^iPSC-CM is minor compared to the difference between iPSC-CMs derived from different tissues. Regarding the cardiac conduction system, there are 13 DEG genes when comparing iPSC-CMs derived from other tissues; meanwhile, there are only 2 DEGs when comparing ^A^iPSC-CM and ^V^iPSC-CM. This trend coincides with the conduction velocity comparison reported in Table 2.

Fibroblasts were the first, (Takahashi and Yamanaka, 2006), and may still be the most common, somatic cells used to generate iPSCs, but the range of sources has expanded to encompass a wide variety of cell types, including cells from the blood, (Hanna et al., 2008), liver, and stomach; (Aoi et al., 2008); neural stem and progenitor cells; (Kim et al., 2008); keratinocytes; (Aasen et al., 2008); melanocytes; (Utikal et al., 2009); and even renal tubular cells obtained from urine (Zhou et al., 2011). However, the transcriptional heterogeneity of iPSCs generated from different cell types can be remarkably high; for example, more than 1,000 genes were differentially expressed (by > 2-fold) between iPSCs that had been reprogrammed from genetically matched mouse tail tip fibroblasts (TTFs) and splenic B cells (sBCs), or from genetically matched bone marrow–derived granulocytes (BMGs) and skeletal muscle precursors (SMPs) (Polo et al., 2010). Furthermore, although SMP-specific (Integrin B1) and BMG-specific (Lysozyme and Gr-1) marker expression were lower in SMP- and BMG-derived iPSCs, respectively, than in the corresponding lineages of somatic cells, SMP markers were more highly expressed in SMP-iPSCs than in BMG-iPSCs, BMG markers were more highly expressed in BMG-iPSCs than in SMP-iPSCs, and these differences in lineage-specific marker expression were accompanied by consistent changes in the presence of activating and suppressing acetylation and methylation markers in the genes’ promoters.

Ample evidence suggests that epigenetic differences can impact both the efficiency of the differentiation procedure and the function of iPSC-derived cells (Kim et al., 2010; Polo et al., 2010; Kim et al., 2011). When iPSCs were reprogrammed from cardiac progenitor cells (CPC-iPSCs) or dermal fibroblasts (DF-iPSCs) and then differentiated into CMs, cTnT was more commonly expressed by CPC-iPSC-CMs, and spontaneous beating was observed in a more significant proportion of embryoid bodies composed of CPC-iPSC-CMs than in DF-iPSC-CM embryoid bodies; (Sanchez-Freire et al., 2014); beating cells also appeared at an earlier timepoint during the differentiation of CPC-iPSC-CMs, and the CPC-iPSC-CMs were more electrophysiologically mature than DF-iPSC-CMs. (Pianezzi et al., 2020). Notably, when iPSCs that had been reprogrammed from fetal neural stem cells (fNSCs) or dermal fibroblasts were differentiated into neural progenitor cells (NPCs), and equal numbers of each iPSC-NPC population were mixed and injected into rodent brains, the engrafted iPSC-NPC population contained a more significant proportion of fNSC-iPSC-NPCs than DF-iPSC-NPCs ten weeks later, (Hargus et al., 2014), which suggests that the epigenetic memory of iPSCs may also have a role in engraftment. However, differences in CPC-iPSC- and DF-iPSC-CM yield were observed for iPSCs that had been passaged up to 30, but not >40 times before differentiation was initiated; (Sanchez-Freire et al., 2014); thus, epigenetic variations between iPSCs derived from different somatic-cell lineages may decline as the iPSCs undergo repeated mitotic events over an extended period. Also, our results do not contradict epigenetics memory heterogeneity in iPSC-CM. Instead, the results suggest that the epigenetics difference becomes so minor when iPSC-CM were generated from the fibroblast of the same tissue (cardiac) that the electrophysiological difference is negligible.

Overall, the effects of how “epigenetic memory” determines functional characteristics of iPSC-CMs depend on the similarity of the source of origin. Thus, while the epigenetic memory promotes important differences in the differentiation efficiency and function of iPSCs reprogrammed from cardiac and non-cardiac origins (Kim et al., 2010; Sanchez-Freire et al., 2014), this effect is weakened when iPSCs were derived from different sublocations of the same tissue (atrial and ventricular fibroblasts). Together these data suggest that epigenetic memory is a process that promotes major phenotypical differences as more divergent is the origin of the iPSCs.

The results presented in this report indicated that ^A^iPSC and ^V^iPSC can be differentiated into CMs with equal efficiency and that the electrophysiological properties and calcium handling activity of ^A^iPSC-CM and ^V^iPSC-CM are broadly similar. Thus, both ^A^iPSC and ^V^iPSC are suitable sources of hiPSC-CMs for investigations of regenerative cardiac therapy and other applications. Our data also demonstrate that iPSC-CMs generated from heart tissue develop better electrophysiological features than those obtained from skin or kidney tissue, suggesting that imprinting mechanisms established in the tissue of origin are retained during the reprogramming process and participate in mediating the expression profile of the newly differentiated cardiomyocytes. Despite the extended use and efficiency of the differentiation protocol used in this work (Lian et al., 2012), the fact that it generates a mixed population of cardiomyocytes may be considered a limitation of our work. Thus, it would be very interesting to compare our data with those obtained using a chamber-specific cardiomyocyte differentiation protocol in the future.

## Data Availability

The original contributions presented in the study are publicly available. The data presented in this study are deposited in the Gene Expression Omnibus (GEO) repository, accession number GSE221268. The article also partially reused the publicly available data from GEO database accession numbers GSE187308 and GSE94267.

## Supplementary material

Supplementary Figure S1 Characterization of ^A^iPSC. **(A)** Cardiac tissue was obtained from the atrium and ventricle of a male newborn infant patient who underwent open-chest surgery for d-TGA and cultured to induce fibroblast outgrowth. **(B)** Isolated fibroblasts (bar=100 μm) were **(C)** evaluated for expression of the fibroblast-specific markers vimentin (VMT) and TE-7 *via* immunofluorescence staining (bar=20 μm) and then reprogrammed **(D)** into ^A^iPSC and ^V^iPSC *via* transfection with Sendai virus coding for OCT4, SOX2, KLF4, and C-MYC (bar=100 μm). **(E)** Three weeks after transduction, putative hiPSCs were identified *via* live immunofluorescent staining for CD44 and the pluripotency marker Tra1-60 (bar=200 μm) and **(F)** imaged for morphological assessments (bar=100 μm). Representative images of cultured atrial tissue (Panel A), atrial fibroblasts (B–C), and ^A^iPSC (D-F) are displayed. **(G)** Expression of the pluripotency markers OCT4, Nanog, SSEA4, and Sox2 was evaluated *via* immunofluorescence staining in ^A^iPSC and ^V^iPSC. Nuclei were counterstained with DAPI (representative images of ^V^iPSC; bar=100 μm.) **(H)** ^A^iPSC and ^V^iPSC were subcutaneously transplanted into immunodeficient mice and grew to form teratomas over the ensuing 8 weeks; then, the teratoma was excised, sectioned, stained with hematoxylin and eosin, and examined for the presence of all three developmental germ layers: ectoderm (i.e., neural epithelium), mesoderm (i.e., striated muscle), and endoderm (i.e., gut-like epithelium) (representative images for ^A^iPSC teratomas; bar=100 μm). **(I)** Representative figures for purity of ^A^iPSC-CM and ^V^iPSC-CM *via* flow cytometry analysis of cTnT-expression cell on B-Day 12.

Supplementary Figure S2 The enriched gene ontologies and KEGG pathways queried from the list of DEGs in: **(A)** comparison between cardiac-tissue-derived iPSC-CM (^A^iPSC-CM and ^V^iPSC-CM) and skin-tissue-derived iPSC-CM (^S-D30^iPSC-CM and ^S-D90^iPSC-CM); **(B)** comparison between ^A^iPSC-CM and ^V^iPSC-CM. The DEG was input into https://david.ncifcrf.gov, resulting in a list of enriched ontologies, pathways, and enrichment p-values. The enrichment score was calculated by taking the negative base10-logarithm of the enrichment p-value.

Supplementary Figure S3 The cardiac conduction DEGs gene expression among different iPSC-CM samples. This include AKAP9, BIN1, CACNA1G, CACNB2, CAV1, DSP, GJA5, IRX3, NKX2-5, RANGRF, RYR2, and ISL1.

Supplementary Figure S4 No significant difference in gene expression was observed between ^A^iPSC-CM and ^V^iPSC-CM samples for atrial, ventricular, pacemaker cardiomyocyte marker. **(A)** Atrium cardiomyocyte markers, including HEY2 and MYL2, expression between ^A^iPSC-CM and ^V^iPSC-CM samples. **(B)** Ventricular cardiomyocyte markers, including MYL7 and NPPA, expression between ^A^iPSC-CM and ^V^iPSC-CM samples. **(C)** Pacemaker cardiomyocyte markers, including HCN4, TBX3, and GJC1, expression between ^A^iPSC-CM and ^V^iPSC-CM samples.
