# Supplementary figures and images for: Comparative analysis of the cardiomyocyte differentiation potential of induced pluripotent stem cells reprogrammed from human atrial or ventricular fibroblasts
Source: Front Bioeng Biotechnol. 2023 Feb 10;11:1108340. doi: 10.3389/fbioe.2023.1108340 (PMC9950567; doi:10.3389/fbioe.2023.1108340)

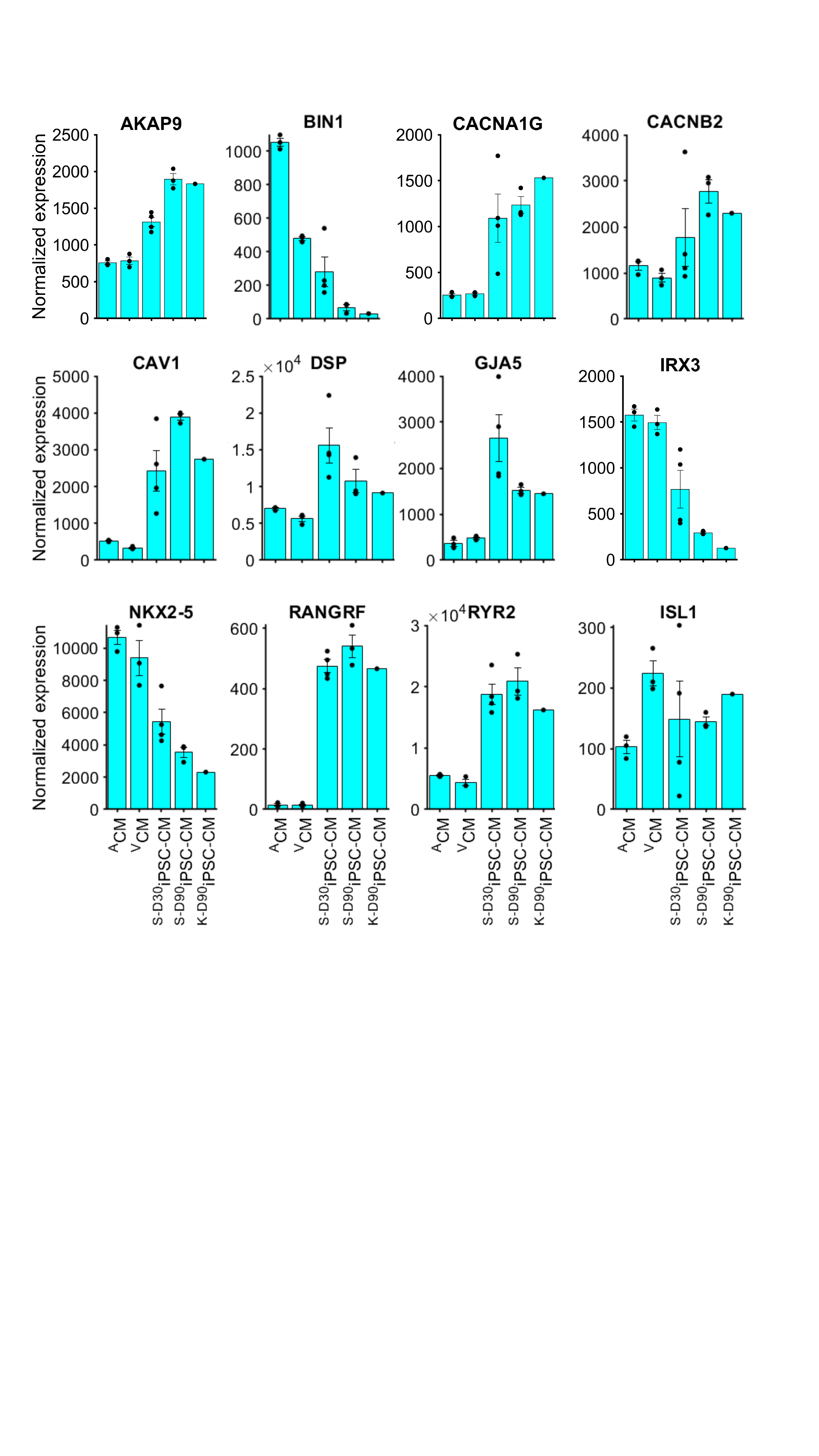

Supplement: Supplementary file 1 [file Image3.JPEG]

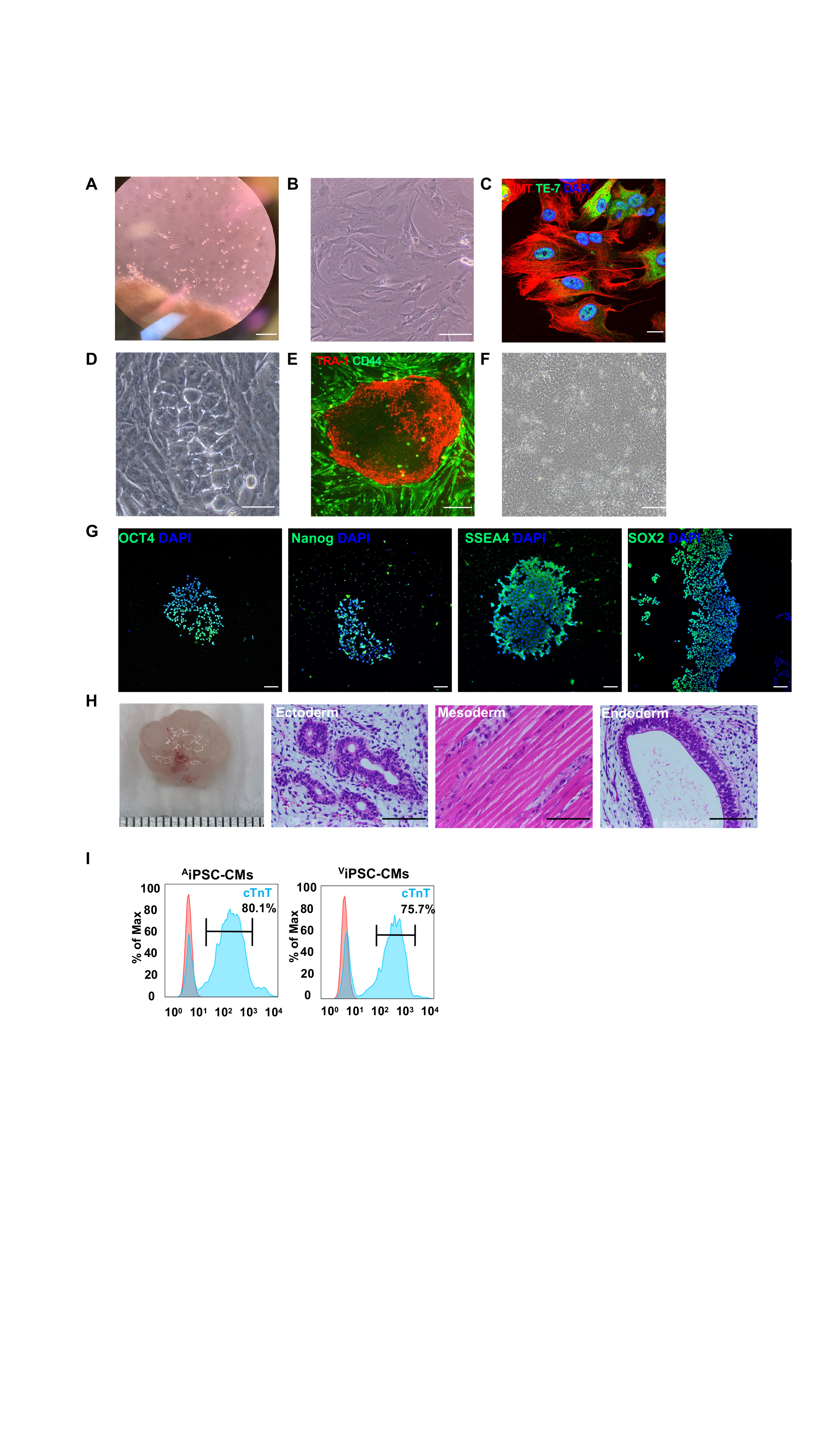

Supplement: Supplementary file 4 [file Image1.JPEG]

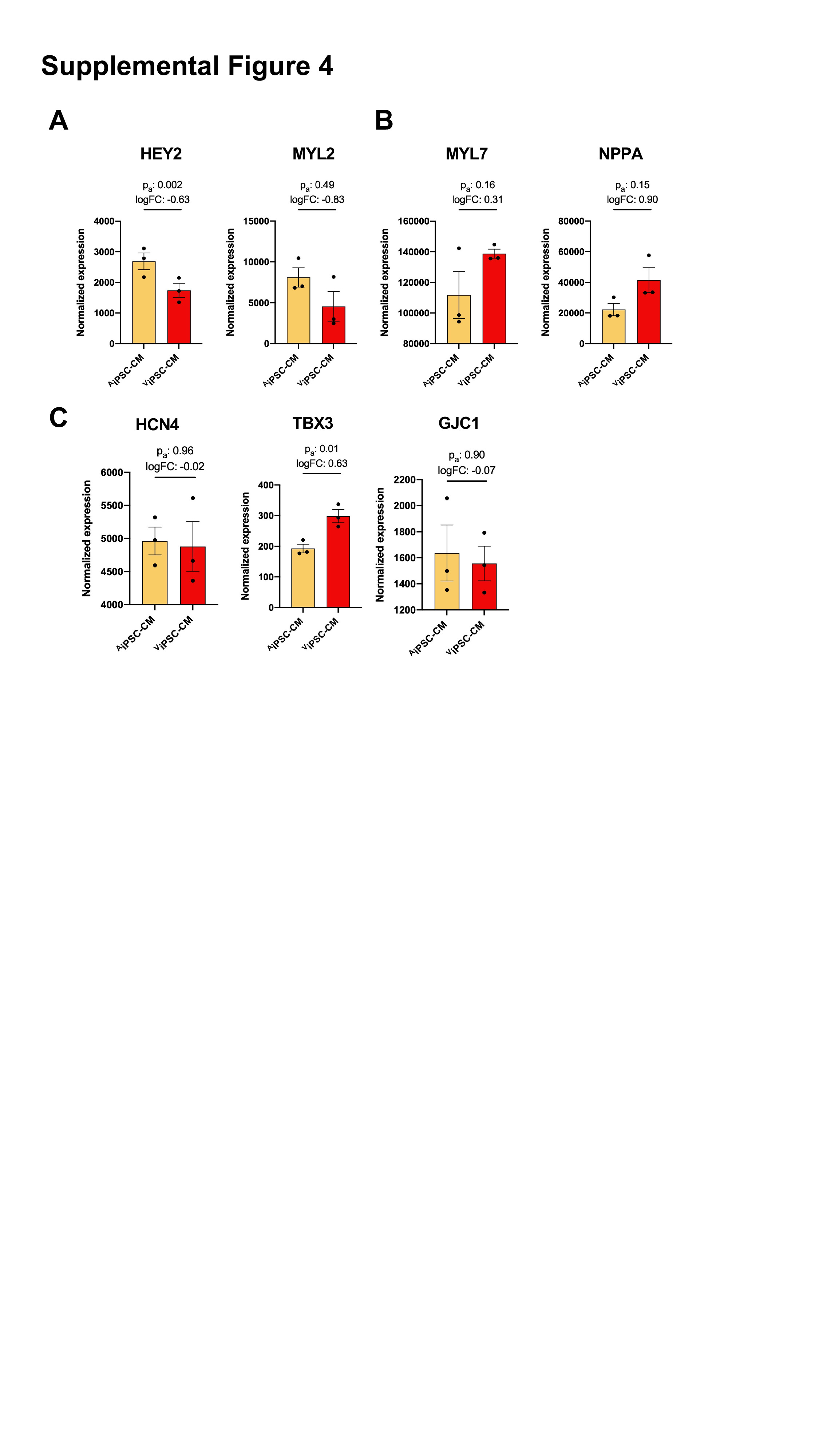

Supplement: Supplementary file 5 [file Image4.JPEG]

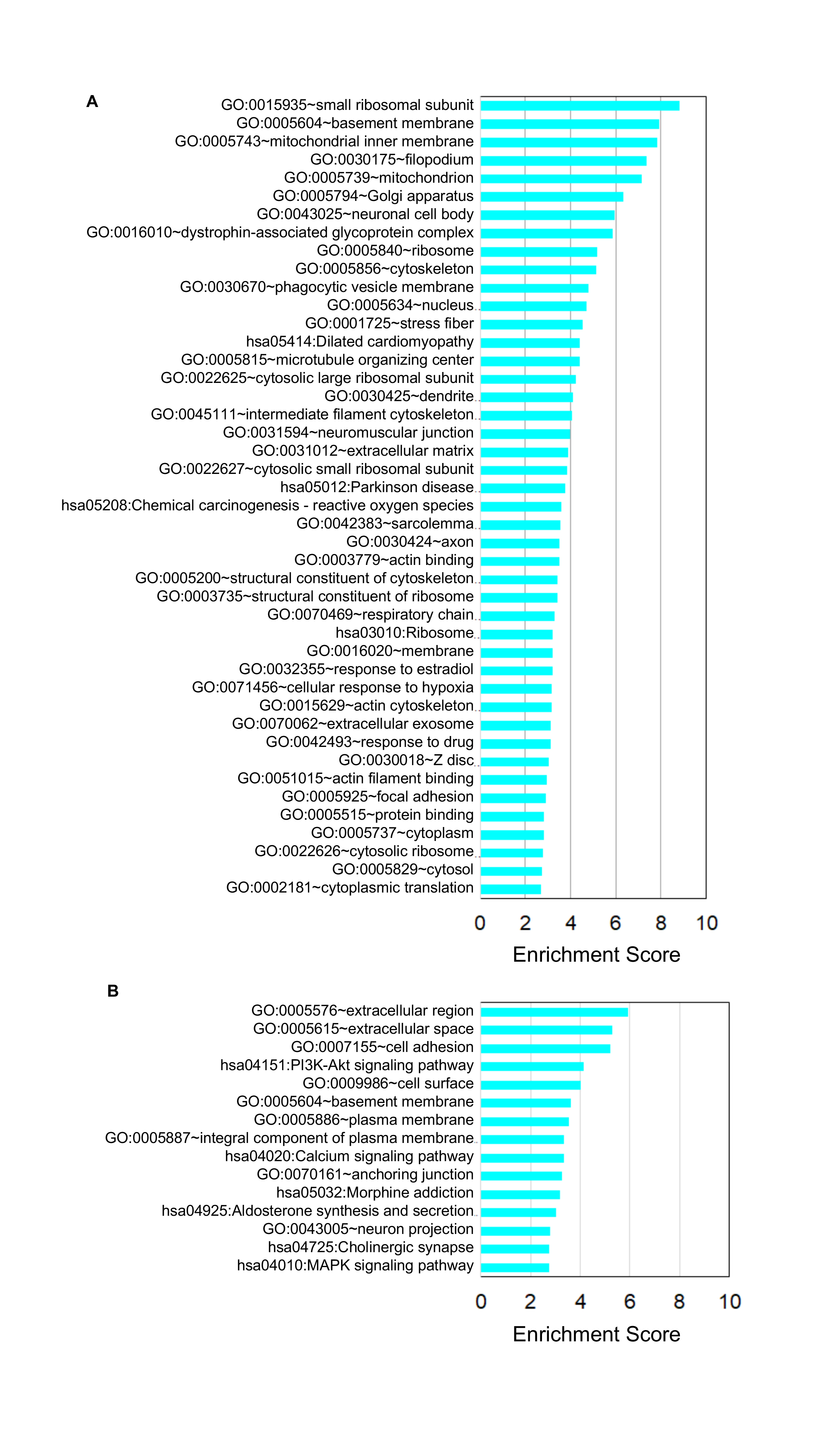

Supplement: Supplementary file 6 [file Image2.JPEG]
