# Supplementary material for: Comparative analysis of the cardiomyocyte differentiation potential of induced pluripotent stem cells reprogrammed from human atrial or ventricular fibroblasts
Source: Front Bioeng Biotechnol. 2023 Feb 10;11:1108340. doi: 10.3389/fbioe.2023.1108340 (PMC9950567; doi:10.3389/fbioe.2023.1108340)
Supplement: Supplementary file 3 [file Table1.pdf]

TABLES

Supplemental Table 1. Antibodies.

Reagent	Manufacturer	Catalog No.	Type	Dilution
Primary Antibodies				
Anti-Vimentin antibody	Abcam	ab92547	Rabbit monoclonal	1:500
Anti-Fibroblasts Antibody, clone TE-7	Millipore	CBL271	Mouse monoclonal	1:100
Anti-Tra-1-60 antibody	Abcam	ab16288	Mouse monoclonal	1:100
Human CD44s Pan Specific Antibody	R&D Systems	BBA10	Mouse monoclonal	1:100
Anti-Oct4 antibody	Abcam	ab19857	Rabbit monoclonal	1:100
Anti-Nanog antibody	Abcam	ab109250	Rabbit monoclonal	1:100
Anti-SOX2 antibody	Abcam	ab97959	Rabbit monoclonal	1:100
Anti-SSEA4 antibody	Abcam	ab16287	Mouse monoclonal	1:100
Secondary Antibodies				
Cy3 AffiniPure Donkey Anti-Rabbit IgG	Jackson ImmunoResearch	711-165-152	Donkey monoclonal	1:200
Cy5 AffiniPure Donkey Anti-Mouse IgG	Jackson ImmunoResearch	715-175-150	Donkey monoclonal	1:200
Fluorescein (FITC) AffiniPure Donkey Anti-Mouse IgG	Jackson ImmunoResearch	715-095-150	Donkey monoclonal	1:200
Fluorescein (FITC) AffiniPure Donkey Anti-Goat IgG	Jackson ImmunoResearch	705-095-003	Donkey monoclonal	1:200
Alexa Fluor 488 AffiniPure Donkey Anti-Goat IgG	Jackson ImmunoResearch	705-545-003	Donkey monoclonal	1:200

Supplemental Table 2. Primers for quantitative RT-PCR.

**Standard gene name**	**Forward 5’-3’**	**Reverse 5’-3’**
OCT4	CAGTGCCCGAAACCCACAC	GGAGACCCAGCAGCCTCAAA
Nanog	TACCTCAGCCTCCAGCAGAT	CTTCTGCGTCACACCATTGC
SOX2	TGGACAGTTACGCGCACAT	CGAGTAGGACATGCTGTAGGT
Brachyury	TGCTTCCCTGAGACCCAGTT	GATCACTTCTTTCCTTTGCATCAAG
MESP1	GAAGTGGTTCCTTGGCAGAC	TCCTGCTTGCCTCAAAGTGT
GATA4	TGCCGTTCATCTTGTGGTAG	CCGACACCCCAATCTCG
NKX2.5	CAAGTGTGCGTCTGCCTTT	CAGCTCTTTCTTTTCGGCTCTA

Supplemental Table 3. The mapping quality of bulk-RNAseq data (12 samples of ^{A}iPSC, ^{V}iPSC, ^{A}iPSC-CM, and ^{V}iPSC-CM groups) was reported by the STAR pipeline.

Supplemental Table 4: the data origin and characteristics of the iPSC and iPSC-CM cell lines, in which Bulk-RNAseq data were integrated and analyzed.

Supplemental Table 5: The statistics of 1081 differentially expressed genes comparing between cardiac-tissue-derived iPSC-CM (^{A}iPSC-CM/^{V}iPSC-CM) and skin-tissue-derived iPSC-CM ($^{S\text{-}D30}$iPSC-CM/ $^{S\text{-}D90}$iPSC-CM). Deseq2 pipeline was used to normalize gene expression. For statistical significance, the fold-change and non-parametric tests were applied.

Supplemental Table 6: The statistics of 124 differentially expressed genes comparing between ^{A}iPSC-CM and ^{V}iPSC-CM. The gene expression was normalized using Deseq2 pipeline, then fold-change and non-parametric tests was applied for statistical significance.
